# Impact of time-history terms on reservoir dynamics and prediction accuracy in echo state networks

**DOI:** 10.1038/s41598-024-59143-y

**Published:** 2024-04-15

**Authors:** Yudai Ebato, Sou Nobukawa, Yusuke Sakemi, Haruhiko Nishimura, Takashi Kanamaru, Nina Sviridova, Kazuyuki Aihara

**Affiliations:** 1 Graduate School of Information and Computer Science, Chiba Institute of Technology, 2-17-1 Tsudanuma, Narashino, Chiba 275-0016 Japan; 2 Department of Computer Science, Chiba Institute of Technology, 2-17-1 Tsudanuma, Narashino, Chiba 275-0016 Japan; 3 Department of Preventive Intervention for Psychiatric Disorders, National Center of Neurology and Psychiatry, 4-1-1 Ogawa-Higashi, Kodaira, Tokyo 187-8551 Japan; 4 Research Center for Mathematical Engineering, Chiba Institute of Technology, 2-17-1 Tsudanuma, Narashino, Chiba 275-0016 Japan; 5 Faculty of Informatics, Yamato University, 2-5-1 Katanama chou, Suita, Osaka 564-0082 Japan; 6 Department of Mechanical Science and Engineering, School of Advanced Engineering, Kogakuin University, 2665-1 Nakano chou, Hachioji, Tokyo 192-0015 Japan; 7 Department of Intelligent Systems, Tokyo City University, 1 choume 28-1 Tamazutsumi, Setagaya, Tokyo, 158-8557 Japan; 8 International Research Center for Neurointelligence, The University of Tokyo Institutes for Advanced Study, The University of Tokyo, 7 choume 3-1 Hongou, Bunkyu ku, Tokyo, 113-8654 Japan

**Keywords:** Leaky integrator ESN, Echo state network, Reservoir computing, Time-series prediction, Time-history terms, Applied mathematics, Nonlinear phenomena, Dynamical systems

## Abstract

The echo state network (ESN) is an excellent machine learning model for processing time-series data. This model, utilising the response of a recurrent neural network, called a reservoir, to input signals, achieves high training efficiency. Introducing time-history terms into the neuron model of the reservoir is known to improve the time-series prediction performance of ESN, yet the reasons for this improvement have not been quantitatively explained in terms of reservoir dynamics characteristics. Therefore, we hypothesised that the performance enhancement brought about by time-history terms could be explained by delay capacity, a recently proposed metric for assessing the memory performance of reservoirs. To test this hypothesis, we conducted comparative experiments using ESN models with time-history terms, namely leaky integrator ESNs (LI-ESN) and chaotic echo state networks (ChESN). The results suggest that compared with ESNs without time-history terms, the reservoir dynamics of LI-ESN and ChESN can maintain diversity and stability while possessing higher delay capacity, leading to their superior performance. Explaining ESN performance through dynamical metrics are crucial for evaluating the numerous ESN architectures recently proposed from a general perspective and for the development of more sophisticated architectures, and this study contributes to such efforts.

## Introduction

**Figure 1:**
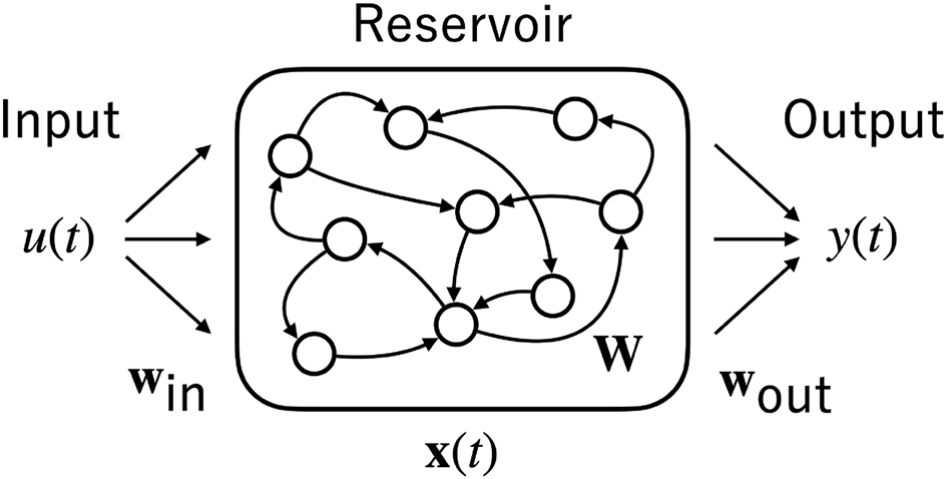
Overview of the structure of echo state network (ESN). Input signal *u*(*t*) is transformed by the input vector $\textbf{w}_{\text {in}}$ and given to all reservoir neurons. The firing state of reservoir neurons is represented by the column vector $\textbf{x}(t)$. The recurrent input to the reservoir neurons is computed as $\textbf{Wx}$ using the weight matrix $\textbf{W}$. The ESN output *y*(*t*) is computed as Eq. (2) using the readout vector $\textbf{w}_{\text {out}}$.

The echo state network (ESN) is a highly efficient machine learning model suitable for processing time-series data^1^. This model has been applied in various engineering applications such as control^2^, speech recognition^3^, motion classification^4^, and network traffic prediction^5^ (reviewed by Tanaka et al.^6^ and Nakajima and Fischer^7^). The ESN comprises three main components: external inputs, a recurrent neural network (RNN) referred to as a reservoir, and a readout layer responsible for extracting spatio-temporal signal responses from the reservoir (refer to the overview of the ESN’s network structure in Fig. 1). In the ESN framework, the connection weights of the reservoir are set random, whereas the readout layer undergoes a learning process^8^. Despite its simplicity, the non-linear responses from the high-dimensional reservoir dynamics, combined linearly by the readout layer, achieve high learning efficiency^9^. This is particularly noteworthy when compared with other RNN-based machine learning models such as backpropagation through time^10^.

To enhance ESN performance, it is crucial to optimise both the structure of the reservoir^11–16^ and the dynamical characteristics of the neurons within it^17^. In the domain of network structure, Kawai et al. studied the impact of small-world topology on signal transfer efficiency within the reservoir^11^. Gallicchio and colleagues introduced a multi-layered reservoir to diversify the time scales of reservoir dynamics^12,13^. Additionally, Iinuma et al. found that parallelisation of reservoir assembly is effective for tasks requiring multi-dimensional inputs^14^. Reservoirs with various neuronal dynamics characteristics, including ESN and liquid state machines (LSMs), have been extensively studied, as shown in the literatures^18,19^. In terms of intrinsic neuronal dynamics, research has highlighted the importance of fine-tuning the neurons’ time-history terms to optimise the time scale of neuronal activity. Leaky integrator ESNs (LI-ESNs) have emerged as prevalent models in this context, enabling the adjustment of neuronal time constants (called as leak rate) to harmonise reservoir dynamics with the input time series’ time scale^20^. Empirical studies have also shown that LI-ESNs outperform ESNs composed of neurons without a leak effect (called as fully-leaky neurons) in terms of time-series prediction capabilities^21^. Furthermore, chaotic neurons, another class of models equipped with multiple decay factors, possess decay coefficients corresponding to external inputs, feedback inputs, and refractory different periods^22^. Unlike leaky integrator neurons, chaotic neurons display spatio-temporally diverse dynamics^23^. Recent findings indicate that ESNs utilising chaotic neurons (chaotic echo state network [ChESN]) also outperform traditional fully-leaky ESNs in time-series predictive performance^24,25^.

Performance improvements due to such reservoir components and the structures can be substantiated through an analysis of reservoir dynamics^26^. The attributes of reservoir dynamics contributing to time-series prediction can be generally categorised into three areas: memory capacity for input signals^27,28^, expressiveness of the output signals^17,29^, and consistency between input and output signals known as echo state property^30–32^. Concretely, the reservoir must preserve relevant information from the input time series for accurate predictions; therefore, the memory capacity is important^27^. Moreover, the expressiveness of the output signals relates to the diversity of reservoir neuronal responses to inputs^17^. Since an ESN’s output is a linear combination of reservoir neuronal behaviours, such diversity is crucial for enhancing the model’s fitting ability^17^. However, overly promoting such diversity can destabilise reservoir dynamics, potentially making the system sensitive to input perturbations or causing long-lasting influence from past inputs, thereby affecting output consistency^31^. Therefore, maintaining an optimal balance between output expressiveness and consistency is essential^33^. Given these considerations, it becomes clear that understanding the reservoir’s dynamical characteristics is vital for optimising performance in time-series prediction tasks.

Previous studies have established that intra-neuronal time-history terms contribute to the time-series prediction performance of ESNs^21,24, 25^. However, to the best of our knowledge, the specific impact of these time-history terms on reservoir dynamics—-and thus on time-series prediction performance—-remains unexplored. These time-history terms can slow down the time scale of reservoir dynamics, facilitating longer retention of input signal information, which is likely to enhance the memory capabilities of the reservoir. In this context, we hypothesisze that the observed performance differences between fully-leaky ESNs and ESNs with time-history terms can be attributed to delay capacity^28^, which serves as a linkage between the time scale and memory performance of the reservoir. In this article, to validate this hypothesis, we aimed to examine the performance of two ESN variants with time-history terms, specifically LI-ESN and ChESN, in two different time-series prediction tasks. In these models, time-history terms of LI-ESN and ChESN correspond to the leak rate and the decay factors, respectively. We also aimed to investigate the memory capabilities of these models’ reservoirs through delay capacity, and we gauge their dynamical diversity and stability using metrics of the covariance rank and consistency.

## Results

Figure 1 shows the structure of an ESN, which is composed of an input layer, a reservoir, and a readout layer. In this architecture, only the weights of the readout layer are trained, which highlights the ESN’s efficiency in handling dynamic inputs. The input signal $u(t) \in \mathbb {R}$ is transformed by the input vector $\textbf{w}_{\text {in}}\in \mathbb {R}^{N_x}$ and then fed into the reservoir. In this study, $N_x$, representing the number of neurons, is set to 100. For clarity, all vectors in this paper, including $\textbf{w}_{\text {in}}$, are presented as column vectors. The reservoir’s state is represented by the vector $\textbf{x}(t)\in \mathbb {R}^{N_x}$, reflecting the neuron firing patterns and playing a crucial role in the network’s processing capability. The recurrent inputs within the reservoir are calculated using the weight matrix $\textbf{W}\in \mathbb {R}^{N_x{\times }N_x}$, represented by $\textbf{Wx}$, facilitating the network’s ability to recognise complex temporal patterns. The output $y(t)\in \mathbb {R}$ is generated using the readout vector $\textbf{w}_{\text {out}}\in \mathbb {R}^{2N_x+1}$, as described in Eq. (2). The update equation for the reservoir of fully-leaky ESN is given the following:

1 $$\begin{aligned} \textbf{x}(t+1) = f( \textbf{w}_{\text {in}} u(t+1) + \textbf{W}\textbf{x}(t)). \end{aligned}$$

Here, $f(\cdot )$ is the element-wise activation function, with $f(\cdot )$ defined as the hyperbolic tangent function. The output of ESN $y(t)$ is obtained by $\textbf{w}_{\text {out}}$:

2 $$\begin{aligned} y(t)=\textbf{w}_{\text {out}}^\textsf{T} \begin{bmatrix} \textbf{x}(t) \\ \textbf{x}(t)^2 \\ 1 \end{bmatrix}. \end{aligned}$$

Here, $\textbf{x}(t)^2$ represents the reservoir firing state vector, where each component is squared^34^. This squared term was incorporated in accordance with Carroll’s research^28^.

In this study, we used two types of ESNs whose reservoir neuron models have time-history terms. The first one is LI-ESN. Unlike the fully-leaky ESN, the update equation for the reservoir in LI-ESN has a parameter called the leak rate $\alpha _l\in (0, 1]$, which adjusts the neuronal time constant:

3 $$\begin{aligned} \textbf{x}(t+1) = (1 - \alpha _l ) \textbf{x}(t) + \alpha _l f( \textbf{w}_{\text {in}} u(t+1) + \textbf{W}\textbf{x}(t)). \end{aligned}$$

When the leak rate $\alpha _l = 1$, it becomes the update equation for fully-leaky ESN. The second one used in this study is ChESN. The chaotic neurons^22,23^ have three internal states corresponding to external inputs, feedback inputs, and refractory periods $\varvec{\xi }(t), \varvec{\eta }(t), \varvec{\zeta }(t) \in \mathbb {R}^{N_x}$ and three decay coefficients $k_e, k_f, k_r \in [0,1)$ corresponding to three internal states:

4 $$\begin{aligned} \begin{aligned} \textbf{x}(t+1)&=f(\varvec{\xi }(t+1)+\varvec{\eta }(t+1)+\varvec{\zeta }(t+1)), \\ \varvec{\xi }(t+1)&=k_e \varvec{\xi }(t)+\textbf{w}_{\text {in}}u(t+1), \\ \varvec{\eta }(t+1)&=k_f \varvec{\eta }(t)+\textbf{W}\textbf{x}(t), \\ \varvec{\zeta }(t+1)&=k_r \varvec{\zeta }(t)-\alpha \textbf{x}(t)+\theta . \end{aligned} \end{aligned}$$

Here, $\alpha \in \mathbb {R}$ is the scaling parameter for refractoriness, and $\theta \in \mathbb {R}$ is the threshold. By adjusting the three decay coefficients of the chaotic neurons, the ChESN is capable of exhibiting a diverse type of dynamics especially including chaotic dynamics. It is important to note that although this model is named ‘chaotic’, the use of chaotic neural networks as reservoirs does not inherently mean utilising chaotic behaviour. Indeed, for optimal reservoir performance, it is often more effective to avoid chaotic behaviour to ensure consistent input-output relationships. Furthermore, to minimise the cost of parameter optimisation in this study, the parameters $k_e$, $\alpha$, and $\theta$ of the ChESN were fixed. Specifically, $k_e=0.01$, $\alpha =0.9$, and $\theta =0$ were set. Moreover, ChESN can be simplified to a form similar to LI-ESN, resulting in comparable performance (refer to Supplementary Note 2 of the Supplementary Materials). Here, the main hyperparameters of fully-leaky ESN are the input scaling parameter $s_{\text {in}}$ in the input layer:

5 $$\begin{aligned} \textbf{w}_{\text {in}} = s_{\text {in}} \textbf{w}^{\text {init}}_{\text {in}} \end{aligned}$$

and the spectral radius of the reservoir coupling weight matrix $\rho (\textbf{W})$, adjusted to *r* by

6 $$\begin{aligned} \textbf{W} = r \frac{\textbf{W}^{\text {init}}}{\rho (\textbf{W}^{\text {init}})}. \end{aligned}$$

Here, $\textbf{w}^{\text {init}}_{\text {in}}\in \mathbb {R}^{N_x}$ is a random vector, and $\textbf{W}^{\text {init}}\in \mathbb {R}^{N_x{\times }N_x}$ is a random sparse matrix with a connectivity rate of 0.1, with elements generated from a uniform distribution in the range $[-1, 1]$. $\rho (\cdot )$ represents the largest eigenvalue. By adjusting these parameters, the reservoir dynamics was optimised for the target task^8^. In addition to these parameters, the time-history terms (in LI-ESN, the leak rate; in ChESN, the decay coefficients) are also adjusted as hyperparameters.

**Figure 2:**
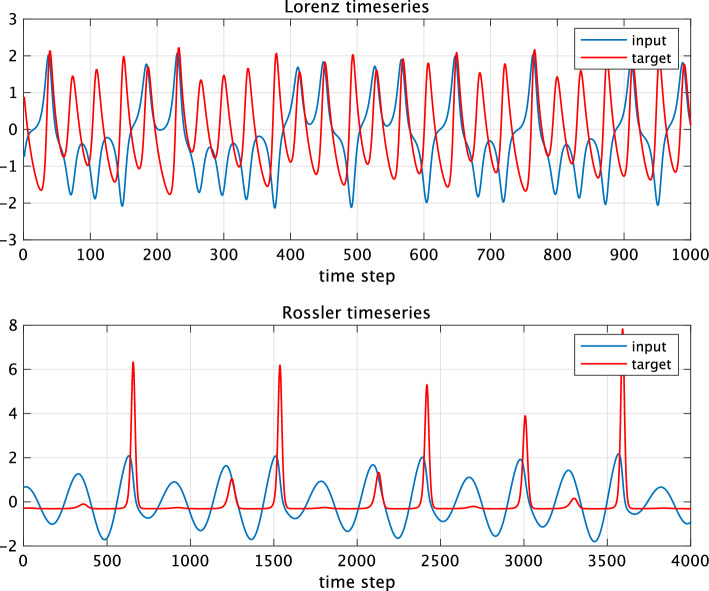
Plot of the time-series data used for prediction task. The blue line represents the input signal, whereas the red line indicates the target output. The tasks predict the *z*-component based on the *x*-component for both the Lorenz and Rössler systems^35,36^. To generate the time series, the fourth-order Runge-Kutta method, with a time step of 0.02, was used for the Lorenz system, whereas for the Rössler system, a time step of 0.3 was used. These time steps correspond to one step of the input signal and the update of the reservoir dynamics.

**Figure 3:**
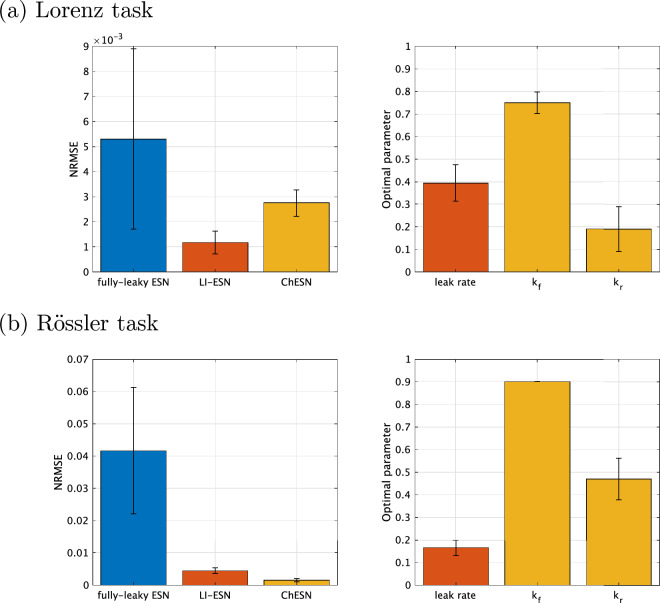
Time-series prediction performance at optimal parameters obtained through grid search. The performance metric is the normalised root mean square error (NRMSE) between the target output and the predictions. The main hyperparameters affecting the performance of the reservoir in each model, namely the spectral radius and input scaling, are set to values that minimise the average NRMSE across 10 trials with varying seed values for LI-ESN and ChESN. For LI-ESN and ChESN, the spectral radius and input scale are fixed at the optimal values, and the time-history term that yielded the lowest NRMSE for each seed value is adopted (i.e., $\alpha _l$ in LI-ESN and $k_f$, $k_r$ in ChESN). For simplicity, some parameters of ChESN are fixed without grid search ($k_e=0$, $\alpha =0.9$, $\theta =0$). Regarding the fully-leaky ESN, the spectral radius and input scaling were optimized for each seed value. Error bars represent the standard deviation across the 10 trials.

**Figure 4:**
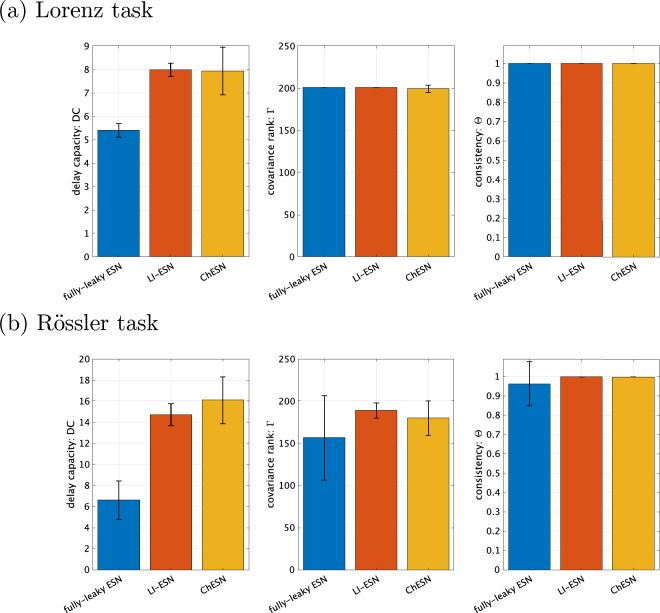
Dynamic indices of high-performing reservoirs. This figure presents the dynamic indices of reservoirs with optimal performance in Fig. 3. Specifically, it illustrates the delay capacity along with metrics for reservoir dynamics diversity (covariance rank) and stability (consistency). Error bars represent the standard deviation across the 10 trials.

**Figure 5:**
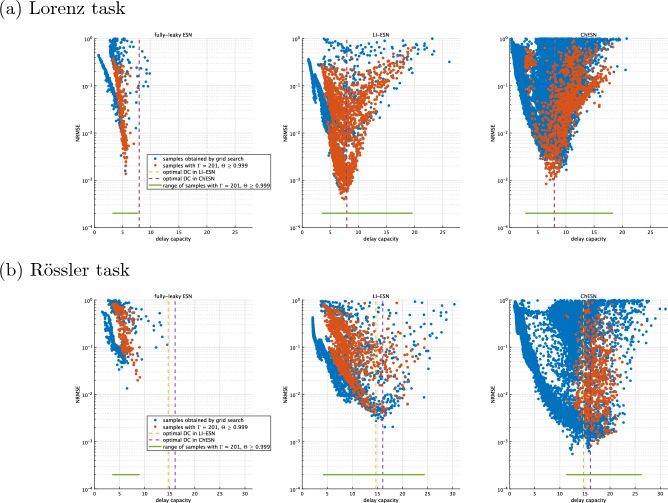
Correspondence between time-series prediction performance and delay capacity. These scatter plots illustrate the relationship between the normalised root mean square error (NRMSE) and delay capacity in time-series prediction tasks for both the Lorenz and Rössler systems. Each point on the scatter plots originates from grid search results, which include the optimal parameters as obtained in Fig. 3. Grid search parameters are shown in Table 1.

**Table 1:** Grid search parameters used in Fig. 5.

(a) Lorenz task		
Model	Input scale	Spectral radius
Fully-leaky ESN	$s_{\text {in}} = 0.1, 0.3, 0.5, \ldots , 1.5$	$\rho (\textbf{W}) = 0.2, 0.4, 0.6, \ldots , 2.0$
LI-ESN	$s_{\text {in}} = 0.1, 0.5, 0.9, 1.3, 1.7$	$\rho (\textbf{W}) = 0.5, 1.0, 1.5$
ChESN	$s_{\text {in}} = 0.3, 0.6, 0.9$	$\rho (\textbf{W}) = 0.1, 0.5, 0.9$
Model	Leak rate	
LI-ESN	$\alpha _l = 0.05, 0.1, 0.15, \ldots , 1.0$	
Model	Decay factor of feedback input	Decay factor of refractory
ChESN	$k_f = 0.05, 0.1, 0.15, \ldots , 1.0$	$k_r = 0.05, 0.1, 0.15, \ldots , 1.0$

(b) Rössler task		
Model	Input scale	Spectral radius
Fully-leaky ESN	$s_{\text {in}} = 0.1, 0.3, 0.5, \ldots , 1.5$	$\rho (\textbf{W}) = 0.2, 0.4, 0.6, \ldots , 2.0$
LI-ESN	$s_{\text {in}} = 0.1, 0.4, 0.7, 1.0, 1.3$	$\rho (\textbf{W}) = 0.6, 1.1, 1.6$
ChESN	$s_{\text {in}} = 0.6, 0.9, 1.2$	$\rho (\textbf{W}) = 0.1, 0.3, 0.5$
Model	Leak rate	
LI-ESN	$\alpha _l = 0.05, 0.1, 0.15, \ldots , 1.0$	
Model	Decay factor of feedback input	Decay factor of refractory
ChESN	$k_f = 0.05, 0.1, 0.15, \ldots , 1.0$	$k_r = 0.05, 0.1, 0.15, \ldots , 1.0$

In all models, both input scale and spectral radius have been subjected to grid search. Additionally, for LI-ESN and ChESN, the time-history terms have also been included in the grid search.

Although previous research has shown that the incorporation of time-history terms can enhance task performance^21,24, 25^, the precise impact of these dynamics remains unclear. To address this, we evaluated the performance implications of time-history terms by utilising indices of reservoir dynamics such as delay capacity, covariance ranks, and consistency. The experiments assessed, first, the enhancement of time-series prediction accuracy through the integration of time-history terms into the ESN; second, the investigation of dynamic indices in reservoirs exhibiting high performance; and third, the examination of the range of delay capacity attainable by each model.

The first experiment involves a comparison of time-series prediction performance. The time-series data used are depicted in Fig. 2. The tasks are to predict the *z*-component based on the *x*-component for both the Lorenz and Rössler systems^35,36^. In the left panels of Fig. 3, which presents the results of the performance comparison, we display the time-series prediction performance at the optimal parameters identified through grid search. This figure reveals that ESNs incorporating time-history terms excel in time-series prediction tasks for both the Lorenz and Rössler systems. These observations confirm that the inclusion of time-history terms in LI-ESN and ChESN enhances predictive performance in time-series tasks.

In the second experiment, we assessed the characteristics of dynamics in the reservoirs with high performance using dynamic indices. Figure 4 illustrates the dynamic indices of the reservoirs, corresponding to the optimal parameters to achieve high accuracy. The figure shows that both LI-ESN and ChESN have higher delay capacities than the fully-leaky ESN. In addition to assessing delay capacity, we concurrently evaluated two other critical metrics: the covariance rank, as a measure of reservoir diversity, and consistency, as a measure of stability. These properties are vital for optimal reservoir performance^17,29, 31, 32^. Across all models, the reservoirs that achieve superior performance shown in Fig. 3 consistently exhibit the highest levels of the covariance rank ($\Gamma = 201$) and consistency ($\Theta \approx 1$), indicating their optimal dynamical diversity and stability.

In high-performance reservoirs with time-history terms, achievement of a delay capacity higher than that of fully-leaky ESNs can be attributed to the presence of these time-history terms. To confirm this, we investigated how delay capacity is distributed in each model under conditions where the covariance rank and consistency are at their maximum values ($\Gamma = 201, \Theta \ge 0.999$). Figure 5 features scatter plots correlating NRMSE in time-series prediction with delay capacity. Each data point on the scatter plots represents a sample obtained through grid search, including the optimal parameters shown in Fig. 3 for each respective model. In Fig. 5, red points indicate samples with a full-rank covariance matrix and consistency values above 0.999. The figure clearly demonstrates that the fully-leaky ESN is unable to achieve the delay capacities observed in the optimal reservoir configurations of LI-ESN and ChESN ($\text {DC} \approx 8$ for the Lorenz task, $\text {DC} \approx 15$ for the Rössler task), as presented in Fig. 3, while also maintaining both diversity and stability. More detailed comparison of the performance involving the case using the other performance metrics and delay capacity in training/validation set among fully-leaky ESN/LI-ESN/ChESN was shown in Supplementary Note 3 of the Supplementary Materials.

**Figure 6:**
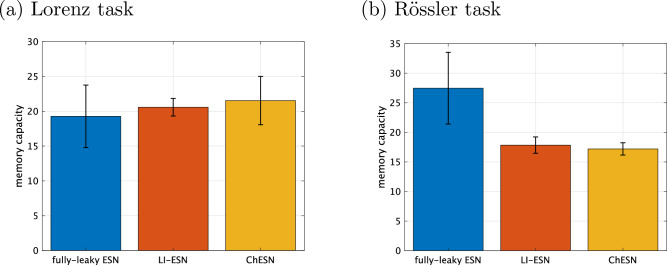
Memory capacity of high-performing reservoirs. This figure presents the memory capacity of reservoirs with optimal performance in Fig. 3. Error bars represent the standard deviation across the 10 trials.

**Figure 7:**
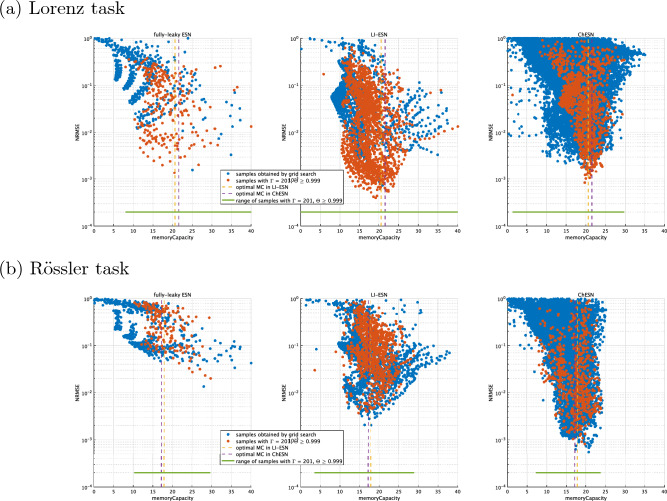
Correspondence between time-series prediction performance and memory capacity. These scatter plots illustrate the relationship between the normalised root mean square error (NRMSE) and memory capacity in time-series prediction tasks for both the Lorenz and Rössler systems. Each point on the scatter plot originates from grid search results, which include the optimal parameters as obtained in Fig. 3. Grid search parameters are shown in Table 1.

Moreover, we presents the evaluation results using memory capacity, a metric more commonly used than delay capacity for measuring the memory of reservoirs. Figure 6 shows the memory capacity in high-performance reservoirs, with parameters identical to those in Fig. 3. Despite the observed performance differences in the Lorenz task, as illustrated in Fig. 3, memory capacity differences were not significant. In the Rössler task, although the time-history terms clearly contribute to performance in LI-ESN and ChESN, the fully-leaky ESN’s memory capacity surpasses those of both LI-ESN and ChESN. Furthermore, Fig. 7 illustrates the distribution of memory capacity across different models. The samples in Fig. 7 were obtained through grid search corresponding to Fig. 5. This figure reveals no substantial differences in the distribution of memory capacity. These observations indicate that although memory capacity measures a similar aspect of reservoir dynamics as delay capacity, it does not account for the performance improvements associated with time-history terms.

## Discussion

Our investigation was primarily aimed at understanding the impact of introducing time-history terms on reservoir dynamics and performance in time-series prediction tasks. Across three different experiments, we validated our hypothesis that the incorporation of time-history terms enhances the delay capacity of the reservoir, thereby improving its performance in time-series prediction. Specifically, the first experiment demonstrated that ESNs with time-history terms outperform those without them in the time-series prediction tasks for both the Lorenz and Rössler systems. The second experiment indicated that high-performing reservoirs tend to exhibit high delay capacities as well as dynamical diversity and stability, as reflected in their covariance ranks and consistency. Finally, the third experiment showed that fully-leaky ESNs are suboptimal in maintaining a high delay capacity while also ensuring stability and diversity. These findings collectively provide evidence that the integration of time-history terms in ESNs significantly augments their capabilities in handling complex time-series tasks.

The performance differences between fully-leaky ESNs and those with time-history terms were analysed from the viewpoint of dynamical characteristics. LI-ESN and ChESN can achieve a broad spectrum of delay capacities, in addition to offering dynamical diversity and stability, as depicted in Fig. 5. Furthermore, despite using different neuron models, LI-ESN and ChESN reach peak performance at similar delay capacity values. These results are consistent with Carroll’s research, which suggests the existence of an optimal level of a reservoir’s memory to retain input information, dependent on factors such as the time scale of the input signal^28^: this optimal memory might correlate with the time scale of the input time series^28^. In our results, for the Rössler task, a greater delay capacity is essential, likely because its time series unfolds at a slower rate than that of the Lorenz system (see Fig. 2). Specifically, the Rössler task necessitates a larger value for optimal performance than the Lorenz task—approximately 8 for Lorenz and about 15 for Rössler, as illustrated in Fig. 4. Although the fully-leaky ESN’s reservoir achieves the maximum covariance rank and consistency, it falls short of the optimal delay capacity range for both tasks. This shortfall is particularly significant in the case of the Rössler task (refer to Fig. 5). These findings indicate that the primary factor in the disparity of performance between fully-leaky ESN and ESN with time-history terms is caused by the range of delay capacity that can be realised.

We analysed why memory capacity failed to align with performance outcomes. Figures 6 and 7 reveal that memory capacity was inadequate in explaining the performance discrepancies in time-series prediction between fully-leaky ESN and ESNs with time-history terms (LI-ESN and ChESN). This limitation may stem from the methodology used to assess memory capacity. Unlike time-series prediction tasks, memory capacity evaluation employs a random time series as the input. LI-ESN and ChESN, with their time-history terms acting as low-pass filters, tend to fail to respond to the fast frequency components of these random series. This oversight leads to an underestimation of LI-ESN and ChESN’s memory performance. In the context of our specific time-series prediction tasks, ignoring these fast frequency components does not lead to deterioration in performance. However, this underestimation becomes more noticeable as the slowing effect of the time-history terms on the reservoir dynamics increases, particularly in the Rössler task compared with the Lorenz task, as evidenced in Fig. 6. Therefore, memory capacity, as assessed in these experiments, proves unsuitable for comparing the memory performances of models affected differently by time-history terms.

The maximum Lyapunov exponent, an indicator of dynamical characteristics distinct from memory performance^37^, was analysed from the perspective of its comparison with the consistency and covariance rank. It was initially intended to be used due to its relatively strong correlation with performance^38^. However, it was considered unsuitable for this study for specific reasons, leading us to substitute it with the covariance rank and consistency. The maximum Lyapunov exponent measures the sensitivity of dynamics to perturbations. Generally, dynamics is considered chaotic when the maximum Lyapunov exponent is greater than 0 and stable when it is negative. It is known that the maximum Lyapunov exponent near 0 (the edge of stability^38^) somewhat corresponds to high reservoir performance. In our time-series predictions, the performance was maximised when the maximum Lyapunov exponent was near 0 (the edge of stability), as shown in Supplementary Fig. S1. This can be interpreted as the reservoir achieving high dynamical diversity at the edge of stability and maintaining consistency in input-output due to the maximum Lyapunov exponent being 0 or below. If the maximum Lyapunov exponent is adopted as a measure of reservoir dynamics characteristics other than memory performance in this study, it is necessary to specify the range of the edge of stability. However, there is no justified method to define this range, making it arbitrary. Additionally, interpreting how the sensitivity of dynamics to perturbations affects performance poses some difficulty. Therefore, the covariance rank and consistency were used to independently measure the diversity and stability of dynamics. Supplementary Fig. S1 is a scatter plot of the maximum Lyapunov exponent and task performance. This figure also shows samples with the maximum covariance rank and maximum consistency. From this figure, it is evident that the maximum consistency is achieved when the maximum Lyapunov exponent is 0 or below, and the maximum covariance rank occurs near 0. Thus, by using the covariance rank and consistency, we could independently assess the dynamical characteristics conventionally gauged by the maximum Lyapunov exponent, which has a strong correlation with performance.

This study has limitations. This study assessed the memory capability, diversity, and stability of reservoir dynamics as aspects influencing time-series prediction performance. However, even with optimal indices, a significant variance in time-series prediction performance persists, as illustrated in Fig. 5. This indicates that there are dynamical properties related to performance that were either not evaluated or not adequately evaluated in this study. Potential areas include non-linearity and synchronicity with the input signal. Moreover, evaluating the diversity of dynamics corresponding to the input time series’ time scale may prove beneficial. To enhance the explainability of the performance of ESNs with time-history terms, future research should focus on exploring these dynamical properties and developing more proper dynamical metrics. Furthermore, investigating whether the findings of this study can be applied not only to simple single-layer reservoirs but also to reservoirs of various structures, such as multi-reservoirs^15^ or self-modulated RC^16^, is also a challenge. Additionally, when applying our findings to models with spiking neurons models in LSM^18,19^, it is crucial to highlight the challenges, because the delay capacity used in our study may not be directly applicable in the case with spiking neurons. Therefore, developing alternative metrics for assessing similar characteristics is imperative. Furthermore, recently, the application of fuzzy computing to RNN has been proposed; therefore such application to the ESNs with time-history terms is an important issue^39^. In addition, the introduction of time-history terms complicates the optimisation of hyperparameters, making it important to evaluate using more efficient optimisation methods proposed in recent years^40^, rather than the grid search used in this study. Moreover, although this study focused on evaluating performance in time-series prediction, exploring the effects of time-history terms in other tasks, such as time-series classification^41^, could also be beneficial. In classification tasks, for instance, the time-history effect, which retains rich information of previous states, could relate not only to the performance but also to the rapidity of recognising transitions in the classified time series.

## Conclusion

In this study, we investigated whether the differences in time-series prediction performance between fully-leaky ESN and ESNs with time-history terms could be explained by delay capacity. Through comparative experiments using LI-ESN and ChESN, we discovered that the limited range of delay capacity achievable by fully-leaky ESN, while simultaneously maintaining the diversity and stability of reservoir dynamics, can account for this performance difference. Clarifying the relationship between reservoir dynamics characteristics and performance is crucial for validating the appropriateness of new ESN architectures with time-history terms and for devising more sophisticated architectures. This research contributes to such objectives.

## Material and methods

### Learning method

The framework for ESN is shown in Fig. 1. In this study, the update equations for the reservoirs in the models—specifically, fully-leaky ESN, LI-ESN, and ChESN—are presented in Eqs. (1), (3), and (4). All other elements of the ESN, excluding the update equations, are common across all models (such as the weights generated for each seed value, the method for calculating model output, and the training procedure for the readout weights).

Training is conducted according to ridge regression for the readout weight $\textbf{w}_{\text {out}}$:

7 $$\begin{aligned} \textbf{w}_{\text {out}}=(\mathbf {\Omega }^\textsf{T}\mathbf {\Omega }+\beta _{\text {ridge}} \textbf{I}_{(2N_x+1) \times (2N_x+1)})^{-1}\mathbf {\Omega }^\textsf{T}\mathbf {y}^{\text {target}}. \end{aligned}$$

In this equation, $\beta _{\text {ridge}}$ represents the regularisation term, which helps to prevent overfitting by penalising large weights. $\textbf{I}_{(2N_x+1) \times (2N_x+1)}$ denotes the identity matrix of size $(2N_x+1) \times (2N_x+1)$. In this ridge regression, the objective is to minimise the squared error between the target output $\textbf{y}^{\text {target}} \in \mathbb {R}^{T_{\text {train}}}$:

8 $$\begin{aligned} \textbf{y}^{\text {target}} = \begin{bmatrix} y^{\text {target}}(T_b+1)&y^{\text {target}}(T_b+2)&\dots&y^{\text {target}}(T_b+T_{\text {train}}) \end{bmatrix}^\textsf{T}, \end{aligned}$$

and the model ouput $\textbf{w}_{\text {out}}$ with collected matrix $\mathbf {\Omega }\in \mathbb {R}^{T_{\text {train}}\times (2N_x+1) }$:

9 $$\begin{aligned} \mathbf {\Omega }= \begin{bmatrix} \textbf{x}(T_b+1) &{} \textbf{x}(T_b+2) &{} \dots &{} \textbf{x}(T_b+T_{\text {train}}) \\ \textbf{x}(T_b+1)^2 &{} \textbf{x}(T_b+2)^2 &{} \dots &{} \textbf{x}(T_b+T_{\text {train}})^2 \\ 1 &{} 1 &{} \dots &{} 1 \end{bmatrix}^\textsf{T}, \end{aligned}$$

which stores the state vector $\textbf{x}(t)$, the square of $\textbf{x}(t)$, and the bias term. The inclusion of $\textbf{x}(t)^2$ accounts for the even non-linearities of the tanh activation function. Here, $T_b = 2000$ represents the burn-in period to eliminate the effects of the reservoir’s initial state, and $T_{\text {train}} = 10000$ is the training period used for ridge regression. Subsequently, the model output is computed as described in Eq. (2).

### Performance evaluation

ESN’s performance was evaluated on time-series prediction tasks generated from the Lorenz and Rössler systems. The Lorenz system is represented by

10 $$\begin{aligned} dx_l/dt&=l_1(y_l-x_l), \nonumber \\ dy_l/dt&=x_l(l_2-z_l)-y_l, \nonumber \\ dz_l/dt&=x_ly_l-l_3z_l. \end{aligned}$$

Here, $l_1=10$, $l_2=28$, and $l_3=8/3$. The Rössler system is described by

11 $$\begin{aligned} dx_r/dt&=-y_r-r_1z_r, \nonumber \\ dy_r/dt&=x_r+r_2y_r, \nonumber \\ dz_r/dt&=r_3+z_r(x_r-r_4). \end{aligned}$$

Here, $r_1=1$, $r_2=0.2$, $r_3=0.2$, and $r_4=5.7$. To generate the reservoir input time series, the fourth-order Runge-Kutta method, with a time step of 0.02, was used for the Lorenz system, whereas for the Rössler system, a time step of 0.3 was used.

In the Lorenz time-series prediction task, the $x_l$ signal was used as the input time series, and the target output was the $z_l$ signal $(y^{\text {target}}(t)=z_l(t))$. In the Rössler time-series prediction task, the input time series was $x_r$, and the target output was the $z_r$ signal $(y^{\text {target}}(t)=z_r(t))$.

The evaluation metric for time-series prediction tasks is normalised root mean squared error (NRMSE):

12 $$\begin{aligned} \textrm{NRMSE} = \sqrt{\frac{\sum _{t = 1}^{T_{\text {test}}}(y(t)-y^{\text {target}}(t))^2}{T_{\text {test}} \sigma ^2(y^{\text {target}})}}. \end{aligned}$$

Here, *y*(*t*) is the output of the ESN and $\sigma ^ 2 (y^{\text {target}})$ is the variance of the target signal. For the evaluation of the performance, other metrics were used, such as mean square error and mean absolute error. The results used by these metrics are shown in Supplementary Note 3 of the Supplementary Materials.

### Evaluation indices

This section first describes the standard index of reservoir dynamics, memory capacity^27^, and its limitation. Then, as the relatively novel indices, delay capacity^28^, consistency^31,32^, and covariance rank^17^ are explained.

#### Memory capacity

Memory capacity is a measure of reservoir memory performance and has been used since the early stage of ESN research^27^. This measure is given by the performance of the delay task, where the past input signal is used as the target output. In this task, the input signal *u*(*t*) is a random time series generated from a Gaussian distribution with mean 0 standard deviation 1. The target output when the delay is $\tau$ is $y^{\text {target}}(t)=u(t-\tau )$ ($\equiv u_\tau (t)$). The delay task performance $\text {MC}_\tau$ is given by

13 $$\begin{aligned} \text {MC}_\tau =\frac{\text {cov}^2(\textbf{u}_\tau ,\textbf{y})}{\sigma ^2(\textbf{u}_\tau )\sigma ^2(\textbf{y})}, \end{aligned}$$

14 $$\begin{aligned} \textbf{u}_\tau =\begin{bmatrix} u_\tau (T_b+1)&u_\tau (T_b+2)&\dots&u_\tau (T_b+T_{\text {test}}) \end{bmatrix}^\textsf{T}, \end{aligned}$$

15 $$\begin{aligned} \textbf{y}= \begin{bmatrix} y(T_b+1)&y(T_b+2)&\dots&y(T_b+T_{\text {test}}) \end{bmatrix}^\textsf{T}. \end{aligned}$$

Here, $\text {cov}(\cdot )$ is the covariance and $\sigma (\cdot )$ is the standard deviation. $\text {MC}$ is given by a sufficiently large value of $\tau _{\text {max}}$ ($\text {MC}_{\tau _{\text {max}}}\approx 0$). $\text {MC}$ is defined by summation of $\text {MC}_\tau$

16 $$\begin{aligned} \text {MC}=\sum ^{\tau _{\text {max}}}_{\tau =1}\text {MC}_\tau . \end{aligned}$$

$\text {MC}$ is a widely used as a benchmark index but cannot assess the reservoir properties’ input signal dependence^28^.

#### Delay capacity

Delay capacity proposed by Carroll is an input signal-independent measure of memory performance^28^. To evaluate delay capacity, two different sets of reservoir state collection matrices are prepared at two different times, and each is whitened. Let the collection matrix at the reference time be denoted as $\textbf{X}_0 \in \mathbb {R}^{N_x\times T_{\text {dc}}}$:

17 $$\begin{aligned}{} & {} \textbf{X}_0=\begin{bmatrix} \textbf{x}(T_b+\tau _{\text {max}}+1)^{\textsf{T}}-\bar{\textbf{x}}^{\textsf{T}} \\ \textbf{x}(T_b+\tau _{\text {max}}+2)^{\textsf{T}}-\bar{\textbf{x}}^{\textsf{T}} \\ \vdots \\ \textbf{x}(T_b+\tau _{\text {max}}+T_{\text {dc}})^{\textsf{T}}-\bar{\textbf{x}}^{\textsf{T}} \\ \end{bmatrix}^{\textsf{T}}, \end{aligned}$$

18 $$\begin{aligned}{} & {} \bar{\textbf{x}}^{\textsf{T}}=\begin{bmatrix} \bar{x}_1&\,&\bar{x}_2&\,&\dots&\,&\bar{x}_{N_x} \end{bmatrix}. \end{aligned}$$

Here, $T_{\text {dc}}$ is the evaluation period of delay capacity. $\tau _{\text {max}}$ represents the maximum delay time for the reservoir state collection matrix at the other time. $\textbf{X}_0$ is centred using the mean state vector $\bar{\textbf{x}}$ of each neuron. Next, the covariance matrix $\textbf{C}\in \mathbb {R}^{N_x\times N_x}$ of $\textbf{X}_0$ is computed by

19 $$\begin{aligned} \textbf{C}=\frac{\textbf{X}_0\textbf{X}_0^{\textsf{T}}}{T_{dc}}+\beta _{\text {reg}}\textbf{I}_{N_x \times N_x}. \end{aligned}$$

Here, $\textbf{I}_{N_x \times N_x}$ denotes the identity matrix of size $N_x \times N_x$. To prevent $\textbf{C}$ from becoming singular, a regularisation term $\beta _{\text {reg}} = 10^{-10}$ is added. Finally, the whitened reservoir state collection matrix $\tilde{\textbf{X}}_0$ is calculated

20 $$\begin{aligned} \tilde{\textbf{X}}_0=\textbf{V}^{\textsf{T}}\mathbf {X_0}\mathbf {\Sigma }^{-\frac{1}{2}}. \end{aligned}$$

through singular value decomposition of $\textbf{C}=\textbf{U}\mathbf {\Sigma }\textbf{V}^{\textsf{T}}$. To assess the reservoir’s memory performance, a time-delayed reservoir state collection matrix $\textbf{X}_{\tau }(t) = \textbf{X}_{0}(t-\tau )\in \mathbb {R}^{N_x\times T_{\text {dc}}}$ is generated, which is $\tau$ steps delayed from the reference time. A correspondingly whitened matrix, $\tilde{\textbf{X}}_{\tau }$, is also generated. The cross-covariance $\textbf{C}(\tau )\in \mathbb {R}^{N_x\times N_x}$ between these two matrices is then calculated by

21 $$\begin{aligned} \textbf{C}(\tau )=\frac{\tilde{\textbf{X}}_0\tilde{\textbf{X}}_{\tau }^{\textsf{T}}}{T_{dc}}, \end{aligned}$$

and the delay capacity ($\text {DC}$) is subsequently determined as follows

22 $$\begin{aligned} \text {DC}=\frac{\sum ^{\tau _\text {max}}_{\tau =1}\text {Trace}|\textbf{C}(\tau )|}{\tau _\text {max}}. \end{aligned}$$

In this formula, $|\cdot |$ signifies the absolute value symbol.

Delay capacity is associated with how long the correlations in the reservoir states are maintained, linking it to memory performance. Unlike memory capacity, this metric allows for the measurement of memory performance under the conditions of task-specific input signals. Although other indices such as the norm of the variation^42^ exist for assessing the memory performance of a reservoir regardless of the input signal, in Carroll’s research Ref.^28^, the experimental results of the delay capacity seemed relatively aligned with performance compared to those of the memory capacity and the norm of the variation, but this point was not explicitly highlighted within it. Additionally, the optimal value of delay capacity is thought to correspond with the autocorrelation of the input time series^28^.

#### Consistency

Consistency is a measure of generalised synchronisation between non-linear systems and inputs^43^ and can be used as a direct evaluation metric for stability^31,32^.

To obtain consistency, a replica test is performed by obtaining the output *y*(*t*) of a reference reservoir and the output $y'(t)$ of the same reservoir (replica reservoir) with a different initial state. The reference reservoir’s initial state is the zero vector, whereas the *i*-th replica reservoir’s initial state $\textbf{x}^{\text {rep}}_i(0) (i=1,2,\dots ,N_s)\in \mathbb {R}^{N_x}$ is generated from a uniform distribution on $[-1,1]$. In this study, the number of different initial conditions, denoted by $N_s$, is set to 10. The coefficient of determination between the reference reservoir’s output $\textbf{y}_{\text {test}}\in \mathbb {R}^{T_{\text {test}}}$:

23 $$\begin{aligned} \textbf{y}_{\text {test}}=\begin{bmatrix} y(T_b+1)&y(T_b+2)&\dots&y(T_b+T_{\text {test}}) \end{bmatrix}^\textsf{T}, \end{aligned}$$

and the replica reservoir’s output $\textbf{y}'_i\in \mathbb {R}^{T_{\text {test}}}$ at initial state $\textbf{x}^{\text {rep}}_i$ is then calculated by

24 $$\begin{aligned}{} & {} \textbf{y}'_i=\begin{bmatrix} y'_i(T_b+1)&y'_i(T_b+2)&\dots&y'_i(T_b+T_{\text {test}}) \end{bmatrix}^\textsf{T}, \end{aligned}$$

25 $$\begin{aligned}{} & {} y'_i(t)=\textbf{w}_{\text {out}}^\textsf{T}\begin{bmatrix} \textbf{x}^{\text {rep}}_i(t) \\ \textbf{x}^{\text {rep}}_i(t)^2 \\ 1 \end{bmatrix}. \end{aligned}$$

This process is done for $N_s$ initial states, and consistency $\Theta$ is defined by

26 $$\begin{aligned}{} & {} \Theta =\sum ^{N_s}_{i=1}C_i, \end{aligned}$$

27 $$\begin{aligned}{} & {} C_i=\frac{\text {cov}^2(\textbf{y}'_i,\textbf{y}_{\text {test}})}{\sigma ^2(\textbf{y}'_i)\sigma ^2(\textbf{y}_{\text {test}})}. \end{aligned}$$

Here, $C_i$ represents the correlation between the reference output and the $i$-th replica output.

#### Covariance rank

The covariance rank of the reservoir behaviour matrix $\Gamma$ can quantify the diversity of reservoir dynamics^17^. Using the reservoir state collection matrix $\mathbf {\Omega }$ defined in Eq. (9):

28 $$\begin{aligned} \Gamma =\text {rank(}\mathbf {\Omega }^{\textsf{T}}\mathbf {\Omega }\text {)}. \end{aligned}$$

To do this, we used the $\text {rank}(\cdot )$ function in MATLAB. This function calculates the number of singular values that exceed a certain threshold $\epsilon$ $(\epsilon \ll 1)$, which is determined with the floating-point relative accuracy.

### Supplementary Information

Supplementary Information.

## Data Availability

The datasets generated and analysed during the current study, as well as the computer codes, are available from the corresponding author upon reasonable request.
