# Supplementary material for: Impact of time-history terms on reservoir dynamics and prediction accuracy in echo state networks
Source: Sci Rep. 2024 Apr 15;14:8631. doi: 10.1038/s41598-024-59143-y (PMC11018609; doi:10.1038/s41598-024-59143-y)
Supplement: Supplementary file 1 — Supplementary Information. [file 41598_2024_59143_MOESM1_ESM.pdf]

Supplementary material: Impact of Time-History Terms on Reservoir Dynamics and Prediction Accuracy in Echo State Networks

Yudai Ebato[1*], Sou Nobukawa[1,2,3,4], Yusuke Sakemi[4,8], Haruhiko Nishimura[5], Takashi Kanamaru[6], Nina Sviridova[7,8], Kazuyuki Aihara[4,8]

[1*]Graduate School of Information and Computer Science, Chiba Institute of Technology, 2-17-1 Tsudanuma, Narashino, Chiba 275-0016, Japan.
[2]Department of Computer Science, Chiba Institute of Technology, 2-17-1 Tsudanuma, Narashino, Chiba 275-0016, Japan.
[3]Department of Preventive Intervention for Psychiatric Disorders, National Center of Neurology and Psychiatry, 4-1-1 Ogawa-Higashi, Kodaira, Tokyo 187-8551, Japan.
[4]Research Center for Mathematical Engineering, Chiba Institute of Technology, 2-17-1 Tsudanuma, Narashino, Chiba 275-0016, Japan.
[5]Faculty of Informatics, Yamato University, 2-5-1 Katanama chou, Suita, Osaka 564-0082, Japan.
[6]Department of Mechanical Science and Engineering, School of Advanced Engineering, Kogakuin University, 192-0015 Tsudanuma, Hachioji, Tokyo 275-0016, Japan.
[7]Department of Intelligent Systems, Tokyo City University, 1 choume 28-1 Tamatsuzumi, Setagaya, Tokyo 158-8557, Japan.
[8]International Research Center for Neurointelligence, The University of Tokyo Institutes for Advanced Study, The University of Tokyo, 7 choume 3-1 Hongou, Bunkyu ku, Tokyo 113-8654, Japan.

*Corresponding author(s). E-mail(s): s1831701fj@s.chibakoudai.jp;
Contributing authors: nobukawa@cs.it-chiba.ac.jp; yusuke.sakemi@p.chibakoudai.jp; nishimura.haruhiko@yamato-u.ac.jp; kanamaru@cc.kogakuin.ac.jp; nina@tcu.ac.jp; kaihara@g.ecc.u-tokyo.ac.jp;

Supplementary Note 1: Analysis of maximum Lyapunov exponent

Maximum Lyapunov exponent

The maximum Lyapunov exponent λ serves as a metric for measuring orbital instability in dynamical systems [1]. This metric is obtained through several equations, which can vary depending on the system under consideration. For a fully-leaky echo state network (ESN)/leaky integrator ESNs (LI-ESN), the reference orbit $\mathbf{s}_k(t)$ is defined as

$$\mathbf{s}_k(t_0) = \mathbf{x}(T_b + \tau k). \tag{S1}$$

In the case of chaotic echo state networks (ChESN), the system possesses three internal states, $\boldsymbol{\xi}(t)$, $\boldsymbol{\eta}(t)$, and $\boldsymbol{\zeta}(t)$, and the reference orbit is given by

$$\mathbf{s}_k(t_0) = \begin{bmatrix} \boldsymbol{\xi}(T_b + \tau k) \\ \boldsymbol{\eta}(T_b + \tau k) \\ \boldsymbol{\zeta}(T_b + \tau k). \end{bmatrix}. \tag{S2}$$

For each initial condition, the perturbed orbit $\mathbf{s}'_k$ evolves over a time period τ, with t_l indicating the time evolution of the perturbation. It starts from an initial condition obtained by adding a perturbation $\boldsymbol{\delta}_k^{(t_l=0)}$ to the reference orbit $\mathbf{s}_k(t)$:

$$\mathbf{s}'_k(t_0) = \mathbf{s}_k(t_0) + \boldsymbol{\delta}_k^{(t_l=0)}. \tag{S3}$$

The difference between the perturbed and reference orbits after the time evolution, denoted by $\boldsymbol{\delta}_k^{(t_l=\tau)}$, is calculated as

$$\boldsymbol{\delta}_k^{(t_l=\tau)} = \mathbf{s}'_k(t_0 + \tau) - \mathbf{s}_k(t_0 + \tau). \tag{S4}$$

For $k > 1$, this initial perturbation is computed using:

$$\boldsymbol{\delta}_k^{(t_l=0)} = \frac{|\boldsymbol{\delta}_1^{(t_l=0)}|}{|\boldsymbol{\delta}_{k-1}^{(t_l=\tau)}|} \boldsymbol{\delta}_{k-1}^{(t_l=\tau)}. \tag{S5}$$

Here, the first perturbation vector $\boldsymbol{\delta}_1^{(t_l=0)}$ is a vector of ones ($\mathbf{1} = [1, 1, \ldots, 1]^T$) scaled to a small value $|\boldsymbol{\delta}_1^{(t_l=0)}| = \delta_0 = 10^{-5}$:

$$\boldsymbol{\delta}_1^{(t_l=0)} = \delta_0 \frac{\mathbf{1}}{|\mathbf{1}|} \tag{S6}$$

The Lyapunov exponent λ_k for each initial condition is determined based on the growth of this perturbation:

$$\lambda_k = \frac{1}{\tau} \ln \frac{|\boldsymbol{\delta}_k^{(t_l=\tau)}|}{|\boldsymbol{\delta}_k^{(t_l=0)}|}. \tag{S7}$$

(a) Lorenz task

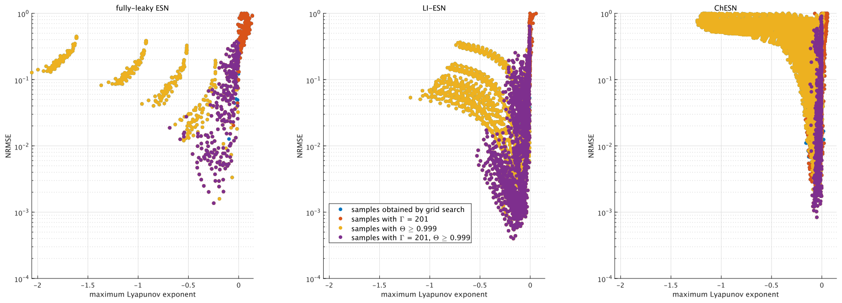

(b) Rössler task

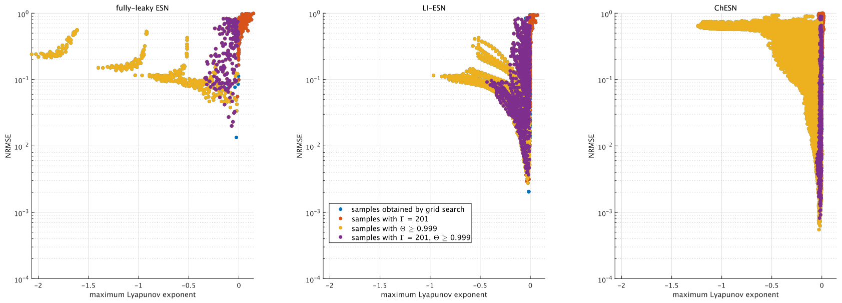

Fig. S1 Correspondence between time-series prediction performance and maximum Lyapunov exponents. This scatter plot illustrates the relationship between the normalised root mean square error (NRMSE) and memory capacity in time-series prediction tasks for both the Lorenz and Rössler systems. Each point on the scatter plot originates from grid search results, which include the optimal parameters as obtained in Fig. 5 in a main text. Grid search parameters are shown in Table 1 in a main text.

Finally, λ is computed by averaging these λ_k values over all K different initial conditions:

$$\lambda = \frac{1}{K}\sum_{k=1}^{K}\lambda_k. \tag{S8}$$

This overall process gives us a measure of the system's sensitivity to initial conditions by capturing the degree of development of the perturbation over time.

Scatter plot of the maximum Lyapunov exponent and performance

Figure. S1 displays the plot of the maximum Lyapunov exponent. This figure clearly shows that the highest performance occurs when the maximum Lyapunov exponent is near zero, indicating the edge of stability [2]. Additionally, consistency typically achieves its maximum value when the Lyapunov exponent is zero or negative. Moreover, the covariance rank tends to be maximised when the maximum Lyapunov exponent is near zero or larger. Notably, at the edge of stability, where the reservoir's

performance is optimised, both the covariance rank and consistency are observed to reach their maximum values.

The maximum Lyapunov exponent is an indicator for evaluating the chaotic characteristic of dynamics, and a system with $\lambda > 0$ exhibits a chaotic state [1]. From the perspective of reservoir computing, the maximum Lyapunov exponent has been used as an index to identify the reservoir dynamics where performance is optimised [2]. The diversity of reservoir dynamics tends to be higher when the maximum Lyapunov exponent is high; however, performance degrades when the Lyapunov exponent exceeds a certain threshold, as the reservoir stability is compromised (i.e., the consistency between the input and output signals is not maintained). Therefore, optimal reservoir dynamics tend to be achieved at high maximum Lyapunov exponents, up to the point just before the stability of the reservoir is lost $\lambda \approx 0$. This point is commonly referred to as the 'edge of chaos', but Carroll argues that the term 'edge of stability' is more appropriate in the context of reservoir computing, as the essential dynamic characteristic is not the system's chaotic nature, but rather the consistency between its input and output signals (echo state property) [2].

Furthermore, achieving optimal performance is not solely dependent on the diversity and stability of the reservoir. Carroll's experiments indicate that the edge of stability does not necessarily correspond to optimal performance, suggesting that there may be other dynamic characteristics that need to be simultaneously fulfilled to achieve optimal performance [2]. According to this finding, in this study, we focus on memory, which is likely related to time-history terms among such performance-related dynamic characteristics.

Moreover, it is not possible to gauge the level of stability and diversity of reservoir dynamics solely from the maximum Lyapunov exponent. In the main text, we introduced metrics for independently measuring dynamic diversity and stability, namely covariance rank [3] and consistency [4, 5], as we need to compare the reservoir dynamics of fully-leaky ESN, LI-ESN, and ChESN.

Supplementary Note 2: Correspondence of simplified ChESN and LI-ESN

We compare the performance of a simple ChESN with LI-ESN. The reservoir update equation for simple ChESN is derived from Eq. (4) in the main text, setting all decay coefficients to a common value ($k = k_e = k_f = k_r$) and scaling the refractory parameter α and threshold θ to zero:

$$\begin{aligned} \mathbf{x}(t+1) &= f(\boldsymbol{\xi}(t+1) + \boldsymbol{\eta}(t+1) + \boldsymbol{\zeta}(t+1)), \\ \mathbf{x}(t+1) &= f(k(\boldsymbol{\xi}(t) + \boldsymbol{\eta}(t) + \boldsymbol{\zeta}(t)) + \mathbf{W}_{\text{in}}\mathbf{u}(t+1) + \mathbf{W}\mathbf{x}(t)). \end{aligned} \tag{S9}$$

Here, by denoting the internal state of the chaotic neuron as $\mathbf{y}_{\mathbf{ch}}(t) = \boldsymbol{\xi}(t) + \boldsymbol{\eta}(t) + \boldsymbol{\zeta}(t)$, Eq. (S9) can be simply expressed as:

$$\begin{aligned} \mathbf{x}(t+1) &= f(\mathbf{y}_{\mathbf{ch}}(t+1)), \\ \mathbf{x}(t+1) &= f(k\mathbf{y}_{\mathbf{ch}}(t) + \mathbf{W}_{\text{in}}\mathbf{u}(t+1) + \mathbf{W}\mathbf{x}(t)). \end{aligned} \tag{S10}$$

(a) Lorenz task

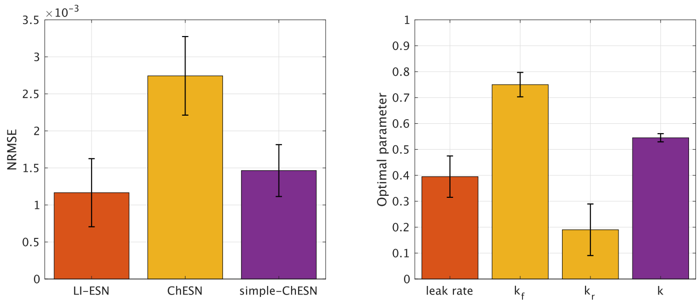

(b) Rössler task

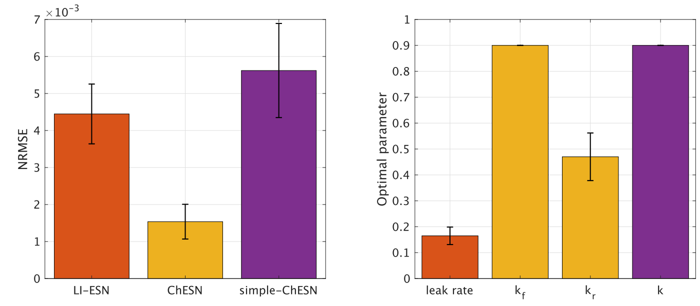

Fig. S2 Time-series prediction performance at optimal parameters obtained through grid search. The performance metric is the normalised root mean square error (NRMSE) between the target output and the predictions. The main hyperparameters affecting the performance of the reservoir in each model, namely the spectral radius and input scaling, are set to values that minimise the average NRMSE across 10 trials with varying seed values. For LI-ESN, ChESN, and simple ChESN (simplified version of ChESN), the spectral radius and input scale are fixed at the optimal values, and the time-history term that yielded the lowest NRMSE for each seed value is adopted (i.e., α_l in LI-ESN, k_f, k_r in ChESN, and k in simple ChESN). For simplicity, some parameters of ChESN are fixed without grid search ($k_e = 0$, $\alpha = 0.9$, $\theta = 0$). Error bars represent the standard deviation across the 10 trials.

Figure. S2, using the same method as Fig. 3 in the main text, shows the time-series prediction performance of the reservoir. From this figure, it is evident that simple ChESN, while not matching, exhibits similar performance values to LI-ESN. Moreover, when comparing simple ChESN with ChESN, simple ChESN outperformed in the Lorenz task.

The inferior performance of simple ChESN compared with that of LI-ESN stems from the different application of time-history terms. Unlike LI-ESN, simple ChESN has decay coefficients in the neuron's internal state, making it more prone to values greater than 1 or less than -1, leading to saturation in the reservoir's firing state due to the tanh activation function. This saturation likely had a negative impact on performance.

Evaluation of the performance difference between simple ChESN and ChESN showed that in the Rössler task, ChESN performed better. This result seems obvious, given that simple ChESN adjusts only k, whereas ChESN adjusts both k_f and

k_r. However, despite such differences in the number of adjustment parameters, simple ChESN outperformed ChESN in the Lorenz task. This could be attributed to the difference in the refractory scaling parameter ($\alpha = 0$ in simple ChESN, and $\alpha = 0.9$ in ChESN). The setting of $\alpha = 0.9$ was adopted from our previous study, which achieved high performance in Mackey-Glass time-series prediction [6]. The Mackey-Glass time series used, generated with a time delay term $\tau = 32$, exhibited a slow time scale, similar to the Rössler task in this study. This suggests that a higher α may be necessary for slow time scale time-series data.

Supplementary Note 3: Verification for overfitting and evaluation for performance with the other metrics

We verified overfitting and evaluated performance with the other metrics in the optimised fully-leaky ESN, LI-ESN, and ChESN. As shown in Fig. 3 in the main text, specifically, to determine whether overfitting has occurred, we compared the performance on the training and validation sets (test data) using multiple metrics, i.e., mean squared error (MSE) and absolute error, other than NRMSE. Additionally, we conducted a comparison of the delay capacity between the training and validation sets, which plays a significant role in this study. As shown in Table 1, the performance and delay capacity in test data did not almost degrade in comparison with training data, suggesting that overfitting has not occurred.

Table 1 Comparison of the time-series prediction performance and delay capacity of the fully-leaky ESN, LI-ESN, and ChESN between the training and validation sets, using the parameters from Fig. 3 in the main text. The results presented are the averages from 10 trials with different random seeds, with standard deviations shown in parentheses. The performance and delay capacity (DC) in test data did not almost degrade.

(a) Lorenz task

Model	NRMSE (train data)	NRMSE (test data)
fully-leaky ESN	$5.30 \times 10^{-3}(4.16 \times 10^{-3})$	$6.16 \times 10^{-3}(3.59 \times 10^{-3})$
LI-ESN	$1.27 \times 10^{-3}(5.22 \times 10^{-4})$	$1.17 \times 10^{-3}(4.61 \times 10^{-4})$
ChESN	$3.15 \times 10^{-3}(7.60 \times 10^{-4})$	$2.74 \times 10^{-3}(5.30 \times 10^{-4})$

Model	MSE (train data)	MSE (test data)
fully-leaky ESN	$5.33 \times 10^{-5}(7.66 \times 10^{-5})$	$4.02 \times 10^{-5}(5.83 \times 10^{-5})$
LI-ESN	$1.85 \times 10^{-6}(1.50 \times 10^{-6})$	$1.57 \times 10^{-6}(1.21 \times 10^{-6})$
ChESN	$1.04 \times 10^{-5}(4.93 \times 10^{-6})$	$7.88 \times 10^{-6}(2.83 \times 10^{-6})$

Model	MAE (train data)	MAE (test data)
fully-leaky ESN	$3.52 \times 10^{-3}(2.13 \times 10^{-3})$	$3.42 \times 10^{-3}(2.01 \times 10^{-3})$
LI-ESN	$8.97 \times 10^{-4}(3.78 \times 10^{-4})$	$8.67 \times 10^{-4}(3.50 \times 10^{-4})$
ChESN	$2.25 \times 10^{-3}(5.04 \times 10^{-4})$	$2.10 \times 10^{-3}(4.17 \times 10^{-4})$

Model	DC (train data)	DC (test data)
fully-leaky ESN	5.40(0.29)	5.23(0.23)
LI-ESN	8.15(0.49)	7.76(0.50)
ChESN	7.93(1.01)	7.62(1.06)

(b) Rössler task

Model	NRMSE (train data)	NRMSE (test data)
fully-leaky ESN	$4.42 \times 10^{-2}(1.70 \times 10^{-2})$	$4.16 \times 10^{-2}(1.95 \times 10^{-2})$
LI-ESN	$5.29 \times 10^{-3}(2.49 \times 10^{-3})$	$6.10 \times 10^{-3}(2.61 \times 10^{-3})$
ChESN	$1.19 \times 10^{-3}(3.71 \times 10^{-3})$	$1.54 \times 10^{-3}(4.69 \times 10^{-4})$

Model	MSE (train data)	MSE (test data)
fully-leaky ESN	$2.36 \times 10^{-3}(1.54 \times 10^{-3})$	$2.35 \times 10^{-3}(1.87 \times 10^{-3})$
LI-ESN	$3.57 \times 10^{-5}(3.82 \times 10^{-5})$	$4.91 \times 10^{-5}(4.74 \times 10^{-5})$
ChESN	$1.64 \times 10^{-6}(1.05 \times 10^{-6})$	$2.90 \times 10^{-6}(1.82 \times 10^{-6})$

Model	MAE (train data)	MAE (test data)
fully-leaky ESN	$2.75 \times 10^{-2}(1.02 \times 10^{-2})$	$2.79 \times 10^{-2}(1.21 \times 10^{-2})$
LI-ESN	$3.71 \times 10^{-3}(1.53 \times 10^{-3})$	$4.32 \times 10^{-3}(1.64 \times 10^{-3})$
ChESN	$9.08 \times 10^{-4}(2.83 \times 10^{-4})$	$1.19 \times 10^{-3}(3.95 \times 10^{-4})$

Model	DC (train data)	DC (test data)
fully-leaky ESN	6.41(1.68)	6.28(1.55)
LI-ESN	14.31(1.17)	14.81(1.28)
ChESN	16.01(2.12)	16.66(2.23)

References

[1] Parker, T.S., Chua, L.: Practical Numerical Algorithms for Chaotic Systems. Springer, New York (2012). https://books.google.co.jp/books?id=lHDjBwAAQBAJ

[2] Carroll, T.L.: Do reservoir computers work best at the edge of chaos? Chaos: An Interdisciplinary Journal of Nonlinear Science **30**(12), 121109 (2020) https://doi.org/10.1063/5.0038163 . Publisher: American Institute of Physics. Accessed 2022-05-23

[3] Carroll, T.L., Pecora, L.M.: Network structure effects in reservoir computers. Chaos: An Interdisciplinary Journal of Nonlinear Science **29**(8), 083130 (2019) https://doi.org/10.1063/1.5097686 . Accessed 2022-08-13

[4] Lymburn, T., Khor, A., Stemler, T., Corrêa, D.C., Small, M., Jüngling, T.: Consistency in echo-state networks. Chaos: An Interdisciplinary Journal of Nonlinear Science **29**(2), 023118 (2019). Publisher: AIP Publishing LLC

[5] Jüngling, T., Lymburn, T., Small, M.: Consistency Hierarchy of Reservoir Computers. IEEE Transactions on Neural Networks and Learning Systems **33**(6), 2586–2595 (2022) https://doi.org/10.1109/TNNLS.2021.3119548 . Conference Name: IEEE Transactions on Neural Networks and Learning Systems

[6] Ebato, Y., Nobukawa, S., Nishimura, H.: Effect of Neural Decay Factors on Prediction Performance in Chaotic Echo State Networks. In: 2021 IEEE International Conference on Systems, Man, and Cybernetics (SMC), pp. 1888–1893 (2021). https://doi.org/10.1109/SMC52423.2021.9659012 . ISSN: 2577-1655
